# Response to Letter to the Editor: “A Genome-Wide Pharmacogenetic Study of Growth Hormone Responsiveness”

**DOI:** 10.1210/clinem/dgaa735

**Published:** 2020-11-11

**Authors:** Joel N Hirschhorn, Andrew Dauber, Laura Audi, Sailaja Vedantam, Michael B Ranke, Alexander A L Jorge, Anders Lindberg, Cecilia Camacho-Hübner, Michael P Wajnrajch

**Affiliations:** 1 Division of Endocrinology, Boston Children’s Hospital, and Program in Medical and Population Genetics, Broad Institute, Harvard Medical School, Boston, Massachusetts; 2 Division of Endocrinology, Children’s National Hospital, Washington, DC; 3 Department of Pediatrics, Institut de Recerca (VHIR), Hospital Vall d’Hebron, Centre for Biomedical Research on Rare Diseases (CIBERER), Autonomous University, Barcelona, Spain; 4 University Children´s Hospital, Paediatric Endocrinology, Tübingen, Germany; 5 Unidade de Endocrinologia do Desenvolvimento (LIM42), Hospital das Clinicas da Faculdade de Medicina da Universidade de Sao Paulo, Sao Paulo, Brazil; 6 Pfizer, Data Management, Sollentuna Sweden; 7 Pfizer Inc, Rare Disease, New York

Pierre Bougnères correctly points out the limitations of our genome-wide association study (GWAS) of variants affecting growth hormone (GH) response, all of which we raised in our “Discussion,” including a relatively small sample size for GWAS (1). Our main goal was to carry out the first genome-wide search powered to detect variants with moderate to large effects on GH response, and to test some specific hypotheses. Although our study size is small compared with most GWAS of polygenic traits, it was adequately powered to detect larger effects that have been seen in other pharmacogenetic settings. Furthermore, the sample sizes needed for replication of individual variants that have themselves been proposed to have large effects, such as the *GHRd3* variant, is much smaller than the study sizes needed for comprehensive GWAS discovery, for which effect sizes are more modest and appropriate correction for multiple testing is required. We were also well powered to test the hypothesis that genetically determined height influences GH responsiveness, because we effectively examined a single hypothesis with large effect: A single polygene score of 697 height-associated variants reflects the combined action of all 697 variants (explaining ~16% of the variance in height [2]).

In specific reference to the *GHRd3* variant, although testing this variant was not the main goal of our study, our results still provide additional information on the likely strength of association with GH responsiveness. The meta-analysis cited by Bougnères (3), which encompassed 1680 children with a wide range of diagnoses and ancestries, reported a 0.075 smaller change in height SD score after 1 year in children who were homozygous for the more common (minor allele frequency ~0.72) wild-type (WT) allele, and a decrease of –0.159 in the subsample with GH deficiency. In our study, the SD of the change in height SD score was 0.4, meaning that the effect sizes of the WT allele reported in the meta-analysis corresponds to a *z* score of –0.075/0.4 = –0.188 in all individuals and –0.159/0.4 = –0.40 in individuals with GH deficiency. These effect sizes correspond to approximately 0.9% and approximately 4% of the variance in GH response explained by this one variant. With these effect sizes, we had 62% and 92% power to achieve a *P* value of less than .05 in our sample of 614 children, of whom 276 had GH deficiency. In our primary analysis, European-ancestry–only analysis, and European-ancestry GH deficiency–only analysis, we actually observed slight trends in the opposite direction (*z* scores of 0.024, 0.002, and 0.011, with *P* values of .66, .97, and .89, respectively). None of the 95% CIs around these *z* scores include the effect sizes reported in the meta-analysis, suggesting that the meta-analysis, at a minimum, has overestimated the effect of the *GHRd3* variant.

Of note, in Wassenaar et al (3), the combined meta-analysis *P* values for association with 2 related measures of GH responsiveness ranged from .03 to .004, and the most significant of these was driven largely by the study that first reported the association (4). We and others have previously noted that most candidate gene association studies that used a *P* value of less than .05 as a threshold for declaring significance turned out to be false positives (5-8), which is why the thresholds for declaring significance have since become much more stringent (genome-wide significance, typically *P* less than 5 × 10^–8^ [9]). We also note that if our study, with a total sample size of more than 600 individuals, were added to the meta-analysis in Wassenaar et al, the combined *P* value would move even further away from this threshold of significance. To borrow the metaphor of David and Goliath from Bougnères, a few special candidate gene association studies hit their target, but most turned out to have gone wide of the mark. Although we cannot definitively say that the association with *GHRd3* and GH responsiveness is a false positive, our data strongly indicate that the effect size is not as large as has been previously suggested.

Finally, with respect to control of population substructure, we analyzed European-ancestry individuals separately and included principal components of ancestry in our analysis. The paper referenced by Bougnères (10) describes the effects of a subtle signal of population stratification on tests of selection that integrate information across genome-wide sets of summary association statistics for adult height; subtle residual stratification is unlikely to have affected the replication, or lack thereof, of individual variants in our study.

## Additional Information

***Disclosure Summary:*** J.N.H. received research support from Pfizer Inc for this study and is on the scientific advisory board of Camp4 Therapeutics; A.D. has received consulting fees from OPKO Biologics, Sandoz, Novo Nordisk, Ipsen, and Pfizer; A.A.L.J. received speaker fees from Sandoz and Novo Nordisk; M.B.R. has received speaker fees from Novo Nordisk, Merck Serono, Sandoz, and Mediagnost. M.P.W. and C.C.H. are employees of Pfizer Inc; A.L. was an employee of Pfizer Health AB; and S.V. and L.A. have nothing to declare.
